# Supplementary material for: Targeting the Crosstalk of Immune Response and Vascular Smooth Muscle Cells Phenotype Switch for Arteriovenous Fistula Maturation
Source: Int J Mol Sci. 2022 Oct 10;23(19):12012. doi: 10.3390/ijms231912012 (PMC9570261; doi:10.3390/ijms231912012)
Supplement: Supplementary file 1 [file ijms-23-12012-s001.zip › ijms-1943951-supplementary.pdf]

Targeting the Crosstalk of Immune Response and Vascular Smooth Muscle Cells Phenotype Switch for Arteriovenous Fistula Maturation

Vikrant Rai, Harbinder Singh, and Devendra K. Agrawal *

Figure S1. RT-qPCR for VACM1, CD11b, and CD68 in VSMCs isolated from control and arteries with plaque. Vascular smooth muscle cell (VSMC), control (C), Vascular Cell Adhesion Molecule (VCAM) 1, cluster of differentiation (CD). All data are presented as mean ± standard deviation (SD). $p<0.05$ is considered statistically significant. ****$p<0.0001$.

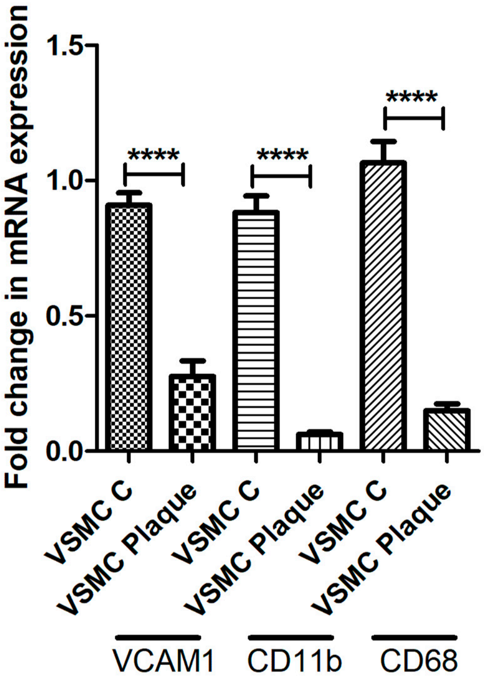

Figure S2. RT-qPCR for the effects of pro-inflammatory cytokines IL-6, IL-1β, and TNF-α on VSMCs phenotype markers. Panel A-RTPCR for α-SMA, TAGLN, and Myh11; panel B- RT-PCR for CK-18, S100A4, CALM2, and ICAM1; and panel C- RT-PCR for KLF4 and CD68. Alpha-smooth muscle actin (α-SMA), transgelin (TAGLN), Myosin Heavy Chain 11 smooth muscle (Myh11), cytokeratin (CK)-18, S100 protein A4 (S100A4), calmodulin 2 (CALM2), Intercellular Adhesion Molecule (ICAM)1, Krüppel-like factor 4 (KLF4). α-SMA, TAGLN, and Myh11 (markers for contractile vascular smooth muscle cells (VSMCs)); CK-18, S100A4, CALM2, and ICAM1 (markers for proliferative VSMCs); and KLF4 (a marker for de-differentiated VSMCs). All data are presented as mean ± standard deviation (SD). $p<0.05$ is considered statistically significant. *$p<0.05$, **$p<0.01$, ***$p<0.001$ and ****$p<0.0001$.

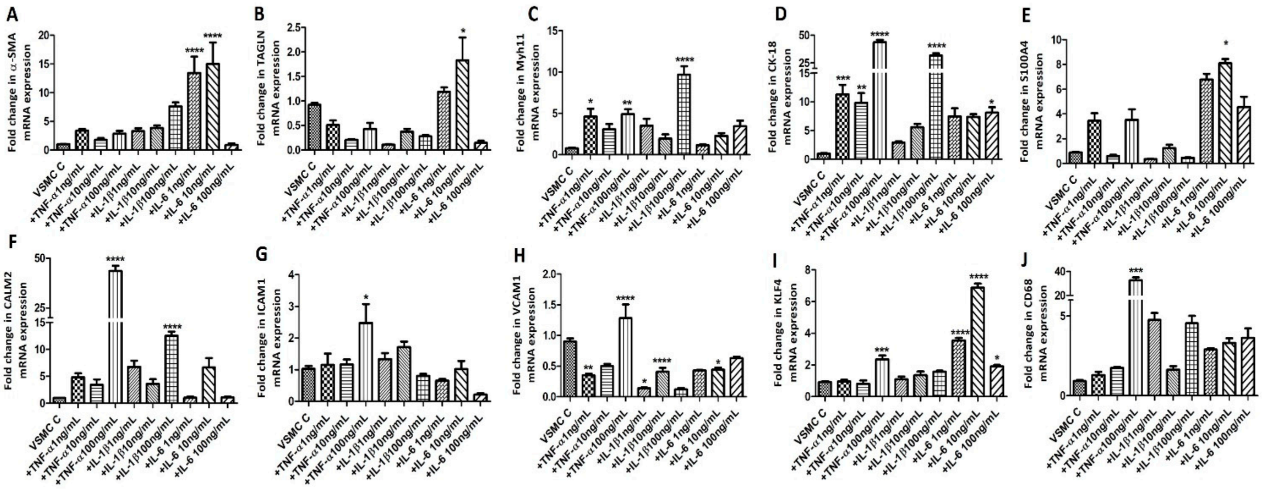

Figure S3. Control artery (panel A) and artery with plaques (panel B) collected after euthanasia. Red circle shows plaque formation and green circle shows adjacent normal artery.

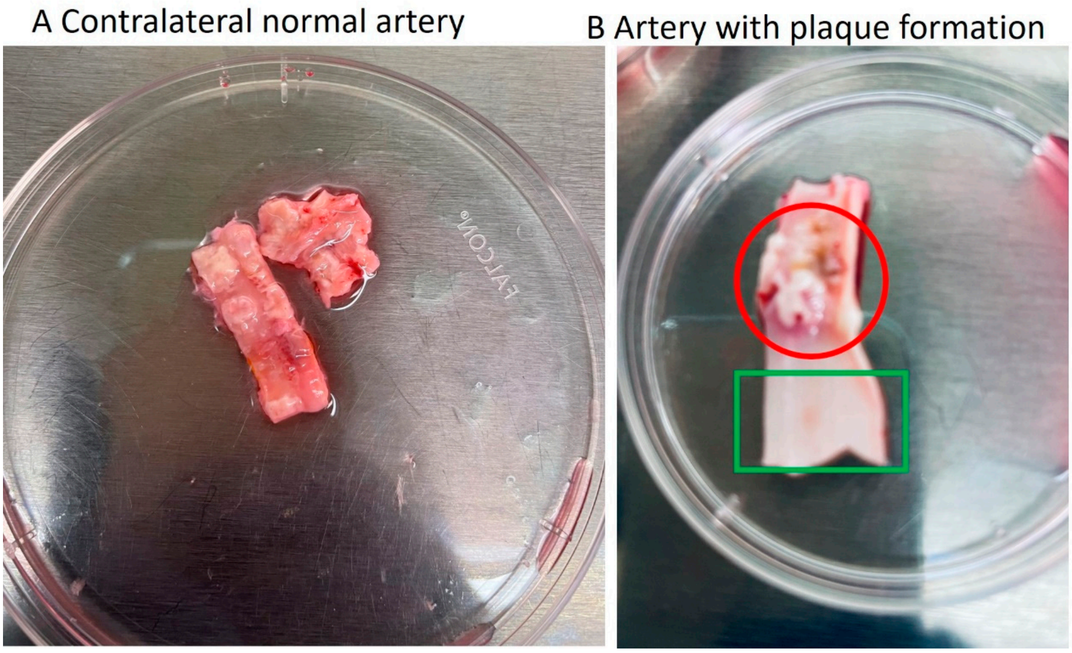

Figure S4. Negative immunofluorescence staining for all antibodies. Alexa Fluor 488 (panel A), Alexa Fluor 596 (panel B), CD68 (panel C), interleukin (IL)-6 (panel D), IL-8 (panel E), tumor necrosis factor (TNF)-α (panel F), α-smooth muscle actin (SMA) (panel G), and IL-1β (panel H).

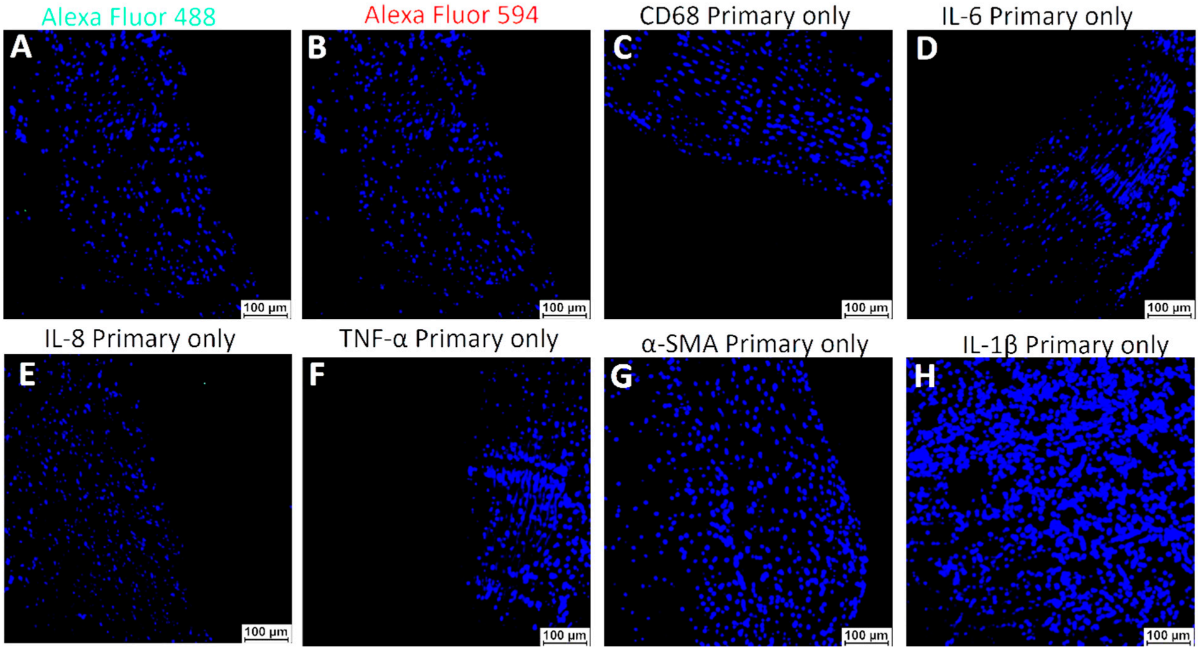

Table S1. Forward and reverse nucleotide sequence of the genes used in RT-PCR. Transgelin (Tagln), myosin heavy chain 11 (Myh11), cytokeratin (CK)-18, S100 protein A4 (S100A4), calmodulin 2 (CALM2), Intercellular Adhesion Molecule (ICAM)1, Krüppel-like factor 4 (KLF4), interleukin (IL), tumor necrosis factor (TNF)-α. α-SMA, TAGLN, and Myh11 (markers for contractile vascular smooth muscle cells (VSMCs)); CK-18, S100A4, CALM2, and ICAM1 (markers for proliferative VSMCs); and KLF4 (a marker for de-differentiated VSMCs), 18S was used as housekeeping gene.

Gene Name		Nucleotide sequence (5'-3')
Tagln	Forward	GGCGATCCCAACTGGTTTAT
Tagln	Reverse	CAGGCCAATGACATGCTTTC
Myh11	Forward	AGTTGGAGATCTGGGACCGA
Myh11	Reverse	TAATGCTGGCTGCCTCGAAA
CK-18	Forward	GGCTCCGCAAGGTCATTGAT
CK-18	Reverse	CAACTCCACGGTCAACCCAG
S100A4	Forward	GTGTCCACCTTCCACAAGTA
S100A4	Reverse	CGGGTCAGCAACTCCTTTA
CALM2	Forward	GACTCCACCAGCCCTTCAAC
CALM 2	Reverse	TAAAGGGAAGGGGCGAACAG
ICAM-1	Forward	CAACCCCCAAGCTGATCTGT
ICAM-1	Reverse	GTGCCTGTAATCTCCCGCTT
VCAM-1	Forward	GCGAGTCCTCCCTGTCTTTC
VCAM-1	Reverse	CGTGGATCTGGTCCCGTTAG
KLF4	Forward	GAGGAGCCAAAGCCAAAGA
KLF4	Reverse	GGTGTGCCTTGAGATGAGAA
CD68	Forward	TCCCAGTGACCAAACCATCC
CD68	Reverse	TTGGAACAGATGCTCACGGA
CD11b	Forward	CAACTTCTCTCTGGTGGGAAAG
CD11b	Reverse	GGGAAACAAGGCTGTGAAGA
TNF-α	Forward	5'-CATCTACCTGGGAGGGGTCT-3'
TNF-α	Reverse	5'-CCAGATAGTCGGGCAGGTTG-3'
IL-6	Forward	5'-TGCAGTCACAGAACGAGTGG-3'
IL-6	Reverse	5'-CAGGTGCCCCAGCTACATTAT-3'
IL-8	Forward	5'-GACCCCAAGGAAAAGTGGGT-3'
IL-8	Reverse	5'-TGACCAGCACAGGAATGAGG-3'
18S	Forward	5'-CCCACGGAATCGAGAAAGAG-3'
18S	Reverse	5'-TTGACGGAAGGGCACCA-3'

Table S2. Antibodies and dilution used for dual immunofluorescence and Western Blot. Interleukin (IL), tumor necrosis factor (TNF)-α, alpha-smooth muscle actin (α-SMA), Krüppel-like factor 4 (KLF4).

**Antibody**	**Catalog no**	**Dilution**
CD68	ab955	1:200
CD68	ab125212	1:100
IL-6	ab6672	1:100
TNF-α	ab6671	1:200
IL-1β	ab9722	1:100
α-SMA	ab5694	1:100
α-SMA	ab7817	1:100
IL-2	sc-133118	1:50
KLF4	NBP2-17070	1:1000
CK-18	MA1-06326	1:500
B-actin	ab8226	1:2000
S100A4	ab197896	1:1000
